# Image statistics of the environment surrounding freely behaving hoverflies

**DOI:** 10.1007/s00359-019-01329-1

**Published:** 2019-04-01

**Authors:** Olga Dyakova, Martin M. Müller, Martin Egelhaaf, Karin Nordström

**Affiliations:** 1 Department of Neuroscience, Uppsala University, Uppsala, Sweden; 2 Neurobiology and CITEC, Bielefeld University, Bielefeld, Germany; 3 Centre for Neuroscience, Flinders University, Adelaide, Australia

**Keywords:** Image statistics, Free flight behavior, Hoverfly, Vision, Modelling

## Abstract

Natural scenes are not as random as they might appear, but are constrained in both space and time. The 2-dimensional spatial constraints can be described by quantifying the image statistics of photographs. Human observers perceive images with naturalistic image statistics as more pleasant to view, and both fly and vertebrate peripheral and higher order visual neurons are tuned to naturalistic image statistics. However, for a given animal, what is natural differs depending on the behavior, and even if we have a broad understanding of image statistics, we know less about the scenes relevant for particular behaviors. To mitigate this, we here investigate the image statistics surrounding *Episyrphus balteatus* hoverflies, where the males hover in sun shafts created by surrounding trees, producing a rich and dense background texture and also intricate shadow patterns on the ground. We quantified the image statistics of photographs of the ground and the surrounding panorama, as the ventral and lateral visual field is particularly important for visual flight control, and found differences in spatial statistics in photos where the hoverflies were hovering compared to where they were flying. Our results can, in the future, be used to create more naturalistic stimuli for experimenter-controlled experiments in the laboratory.

## Introduction

At a glance, natural scenes appear to be extremely complex and to provide more information than biological visual systems could possibly deal with appropriately. Already, von Helmholtz (1867) therefore suggested that animal visual systems could code for such immense information by simplifying the input. About 100 years later it was shown that natural input is more constrained than it appears, in both space and time, and that early visual processing appears to utilize the expected redundancy (e.g., Barlow 1961): Animals with eyes optimize visual information transmission using evolutionary and developmental adaptations to their natural environments.

To understand the behavioral relevance of such coding adaptations it is important to consider the relevant natural environments in which the animals behave. For this purpose, we can use image statistics, which is a method for quantifying the two-dimensional information in a picture. Some image statistics, such as image color, contrast, skewness (Bex and Makous 2002; Kumar and Gupta 2012; Pouli et al. 2011) and entropy, are based on the luminance and color values of a picture’s individual pixels. Entropy can be used to describe the complexity of an image (Redies et al. 2017), where homogenous images with uniform backgrounds and uniform objects have low entropy. When human observers view natural scenes, they tend to shift their gaze to regions with higher entropy (Reinagel and Zador 1999; Renninger et al. 2007; Itti and Baldi 2009). In addition, human reaction time when categorizing images increases with entropy (Mirzaei et al. 2013). Note that high entropy does not imply that an image is more naturalistic, as white noise images, where all adjacent pixels are uncorrelated, have high entropy (Ruderman and Bialek 1994).

Image statistics that take the relationships between the pixels’ positions into account (van der Schaaf and van Hateren 1996) is a valuable and important measure as neighboring spatial locations have highly correlated intensity values in natural input (Simoncelli and Olshausen 2001). A widely used and well-investigated image statistic is the slope constant of the amplitude spectrum, which can be extracted after doing a Fourier transform of the image (Field 1987). On a log–log scale, the rotational average of the amplitude spectra of natural scenes has a $\frac{1}{{f}^{\alpha }}$ shape, with slope constants (alpha) around 1–1.2 (Tolhurst et al. 1992; Bex and Makous 2002; Schwegmann et al. 2014b). When alpha is exactly one, the scene is scale-invariant.

A large body of work suggests that both human and fly peripheral visual systems have evolved to encode natural scenes optimally. For example, Atick and Redlich (1992) showed that the vertebrate retina works as a ‘whitening’ filter of natural input by transforming the 1/*f* amplitude spectrum into a flat spectrum. Similar ‘whitening’ takes place in the fly retina (Laughlin 1981). In more central processing, it has been suggested that orientation tuning, bandwidth tuning and the receptive field structure of the cortical cells in V1 optimally encode natural stimuli with 1/*f* characteristics (Field 1987; Field and Brady 1997). In hoverflies, the inhibition of a higher order visual neuron in the brain is strongest when alpha is close to one (Dyakova et al. 2015) and optic flow-sensitive neurons in the descending nerve cord are also tuned to naturalistic alpha values (Nicholas et al. 2018).

However, even if we can define typical image statistics, there are important differences between scene categories. For example, the amount of man-made structures, the distance between the observer and the scene being depicted, and the level of scene “openness” all have different image statistics, including slope constants (alpha) (Torralba and Oliva 2003; McCotter et al. 2005). Furthermore, within a given scene local image statistics may vary dramatically (Frazor and Geisler 2006; Schwegmann et al. 2014b). To understand the visual coding of natural scenes, we thus need to define natural for a particular animal performing a specific behavior. This is important as most animals perform different behaviors against different backdrops, creating many different “natural” scenes.

Hoverflies are arising as emerging models in insect vision, and it is therefore becoming pertinent to understand their natural environment in more detail. Compared to many other insects, we have a detailed understanding of, e.g., the neural encoding of visual stimuli by single higher order neurons in the dronefly *Eristalis tenax* (Dyakova et al. 2015; Nordström and O’Carroll 2009), of target pursuit behavior in several hoverflies (Collett and Land 1978; Collett and King 1975), and of the optomotor response in *Episyrphus balteatus* (Goulard et al. 2015). Here we focus on the marmalade fly *Episyrphus balteatus*, which is an important commercial pollinator and aphid controller (Sutherland et al. 1999; Martinez-Una et al. 2013) commonly found in gardens, parks or fields across Europe (Gilbert and Owen 1990). In these open environments, they inspect flowers for feeding, seek moisture, and search for oviposition sites and for potential mates (Primante and Dötterl 2010; Verheggen et al. 2008; Goulson and Wright 1998).

As the name implies, hoverflies are characterized by their ability to remain near-stationary mid-air for prolonged periods of time (Collett and Land 1978). *Episyrphus* males set up their hovering territories in sun shafts created by tree branches (Alderman 2008). The resulting ground shade pattern produced by the foliage is often quite striking, especially when compared with the more open fields that the hoverflies may cross in their search for flowers or partners. To understand more about the image statistics that may be relevant to hoverflies we took photos where *E. balteatus* were observed to be freely hovering in the field. As comparison we identified locations where *E. balteatus* hoverflies were flying, as this behavior is performed at a similar elevation. We took photos of the ground over which the hoverfly was performing its behavior and panoramic photographs of the surrounding. We focused on images from the ventral and lateral parts of the visual field as these appear to be particularly important for insect flight control (Linander et al. 2017; 2018; Portelli et al. 2011; Straw et al. 2010). In addition, panoramic images have been used extensively to understand motion vision coding in the insect brain (for hoverfly examples, see e.g., O’Carroll et al. 2011, 2012; Barnett et al. 2010; Straw et al. 2008). From the photos we extracted the slope constant of the amplitude spectrum, as this has been shown to affect the response properties of visual neurons in the hoverfly brain (Dyakova et al. 2015) and ventral nerve cord (Nicholas et al. 2018). In addition, we extracted image entropy, as the intricate shadows created by the sun shafts suggested that the two behaviors could be associated with different levels of image complexity. We found that both the slope constant (alpha) and the entropy were significantly different between photos taken where hoverflies were performing the two behaviors. Our findings highlight that to determine what is natural, one needs to take the relevant behavior into account.

## Materials and methods

### Behavior

We focused on two behaviors of the marmalade hoverfly *Episyrphus balteatus*: hovering and flying. The two different behaviors were manually scored in the field, in Germany close to Freiburg, in the Baden-Württemberg region (red, Fig. 5c), in July when marmalade hoverflies are out in abundance.

A hoverfly was scored as hovering if it was observed to be near-stationary mid-air, often more than 1 m over the ground, for 60 s. Male *Episyrphus balteatus* are excellent hoverers and often remain stationary for longer than this (Ellington and Lighthill 1984). Hoverflies can be flying in many different places and for many different reasons (e.g., for foraging, pursuing territorial intruders or mates; Alderman 2008; Almohamad et al. 2009), and here, in contrast to hovering behavior, the fly was scored as flying if it appeared to be flying from point to point, without stopping or returning to a given starting position. As all hoverflies were identified manually, there is a possibility that our own perceptual bias affected the locations chosen. As hovering is a male-specific behavior, it is likely that all hovering hoverflies were male, whereas the flying hoverflies could have been either male or female.

### Photographs

We took RAW (NEF) format photos of the surrounding panorama and of the ground using a 12 bit full-frame digital single-lens reflex Nikon D700 camera with a resolution of 4256 × 2832 pixels. We manually controlled the focus of the camera to reduce the artificial blurriness which otherwise increases the slope constant (Field and Brady 1997). Photos of the ground were obtained facing the ground from approximately 1 m height, from the location where the hoverfly was observed to be either hovering or flying. The size of these photos corresponded to approximately 1 × 1.5 m, which from 1 m height corresponds to ca. 53° × 80° of the visual field of view.

Panoramic photos were centered on the location where the hoverfly was originally observed to be hovering or flying. The camera was placed on a tripod with a panoramic head, ca 1 m above the ground, using a level. We took 11–13 evenly spaced photos (2832 pixels width × 4256 pixels height) to get the full 360° coverage. The resulting panorama was created with Adobe Photoshop’s photomerge function had an average final size of 14,148 × 3924 pixels, corresponding to 70° × 360° of the visual field of view.

#### Image statistics

For image statistics each RAW (NEF) format photo was converted to grayscale in Matlab (http://www.mathworks.com) using the green channel of the RGB photo, since there are more fly photoreceptors sensitive to this part of the spectrum, with some additional sensitivity of the shorter wavelength photoreceptors (Horridge et al. 1975; Salcedo et al. 1999; Srinivasan and Guy 1990). These images were then converted to double format and gamma corrected (Reinhard et al. 2010). For ground photos, we cropped the images to the central 2832 × 2832 pixel square, which were analyzed separately, corresponding to 53 × 53° of the visual field of view. For the panorama photos we cropped three overlapping 2832 × 2832 pixel squares corresponding to the top, middle and bottom elevations. Each square of these panoramic segments corresponded to 70 × 70° of the visual field of view, with the top segment covering ca 50° over the equator to 20° under it, the middle to ± 35° around the equator, and the bottom to ca 20° degrees over the equator to 50° under it.

To calculate the slope constant, we used our previously described method (Dyakova et al. 2015). Briefly, each grayscale image was first transformed to a Fourier matrix using the Matlab function *fft2*, the zero-frequency component was shifted to the center using the Matlab function *fftshift*, and the amplitude spectrum extracted using the Matlab function *abs*. We next converted the amplitude spectrum to polar coordinates using the Matlab function *cart2pol*, and calculated the rotationally averaged amplitude. The slope constant (alpha) was identified using the Matlab function *polyfit* between 0.1 and 1 cycles per degree (cpd), which corresponded to 5 and 53 cycles per image (cpi) for the ground photos and 4 and 101 cpi for the panoramic segments, similar to previous work (Field and Brady 1997; Dyakova et al. 2015).

To calculate the entropy each photo was first low-pass filtered with a cutoff frequency of 1 cpd to take the hoverfly’s optics into account (Dyakova et al. 2015; Straw et al. 2006), which corresponded to 53 cpi for the ground photos and 101 cpi for the panoramic segments. Entropy was calculated using Matlab’s function *entropy*, which gives the entropy as a scalar value representing the randomness as a quantification of the image texture (Gonzalez et al. 2009):

$$E= -\sum _{k}{p}_{k}{\text{log}}_{2}\left({p}_{k}\right)$$

where *E* is the entropy, *k* is the number of grey levels, and *p*_*k*_ is the probability associated with grey level *k*. The entropy is maximal in the case of a uniform probability distribution (for more details see Annadurai 2007; Eichkitz et al. 2013; Petroni 2014):

$${p}_{k}=\frac{1}{L-1}, \quad {\rm for} \ k \ = \ 0,1,2,\ldots,L-1.$$

### Average skyline vector (ASV) computation

To find the average skyline vector (ASV, Müller et al. 2018), we used the alpha or entropy values calculated from the top, middle or bottom elevation segments for the 11–13 photos contributing to each panoramic surround. Each value was assigned a direction based on the total number of photos contributing to each panorama, so that they together covered the entire 360° azimuthal field of view, e.g., if there were 12 photos, these were placed 30° apart with assigned directions of 0°, 30°, 60° etc., such that the order of directions matched the order of image segments from which the alpha or entropy values were derived (see e.g., the flying example, Fig. 4c). This means that each set of image segments that formed a horizontal (top, middle or bottom) slice of the panorama was represented by a set of 2D-vectors, evenly spaced around a cylinder. The magnitude of each of these vectors was defined by their respective alpha or entropy values, while their direction was based on the position of the image segment from which the alpha or entropy value was derived.

Next, we calculated the absolute sum of these vectors to get one average skyline vector (||ASV||) for each elevation (top, middle, and bottom) for each panorama. If the alpha or entropy values were perfectly balanced in all directions, the magnitude of the summed vector would be 0, thereby indicating perfect symmetry. The larger the variation in alpha or entropy, the more unbalanced the values are in the different directions, creating a larger magnitude of the resulting summed vector (and thus the asymmetry value). For comparison between hovering and flying, we quantified the magnitude of the resulting ASV, irrespective of direction.

### Logistic-regression model

A logistic-regression equation provides the predicted logit of the outcome, which in our case was given as 1 for hovering and 0 for flying, using:

$$\text{ln}\left(\frac{P}{P-1}\right)=a+bx$$

where *P* is the probability of hovering occurring, *x* is the dependent variable (in our case either alpha or entropy), and the coefficients *a* and *b* were obtained using the Matlab function *fitglm*. The distribution of the dependent variables was set to binominal. To get the *x* and *y* coordinates for the receiver operating characteristic (ROC) curve and its area under the curve (AUC) the Matlab function *perfcurve* was used. *X* coordinate relates to sensitivity, which is the proportion of true positives, i.e., in our case the proportion of cases correctly identified by the test as hovering behavior, and *y* coordinate relates to (1—specificity), where specificity is the proportion of true negatives, i.e., in our case the proportion of cases correctly identified by the test as not hovering, i.e., flying (Fawcett 2004):

$$\text{True\,positive\,rate}=\frac{\text{positives\,correctly\,classified}}{\text{total\,positives}}$$

$$\text{False\,positive\,rate}=\frac{\text{negatives\,incorrectly\,classified}}{\text{total\,negatives}}$$

To test the model’s ability to predict hoverfly behavior we obtained an independent set of photos from different locations (blue, Fig. 5c), including Sweden (August, September), Germany (May–August) and Croatia (June). The probability (*P*) of observing a particular behavior in a new photo was extracted from the output of the logistic-regression model using:

$$P=\frac{{e}^{\left(ax+b\right)}}{1+{e}^{\left(ax+b\right)}}$$

where *x* is the image variable (in our case alpha) and *a* and *b* are given by the equation above. Note, that the probability (*P*) here gives “hovering”. Thus, if the investigated photo came from a hovering position, then the probability (*P*) of the correct prediction comes directly. If the photo came from a flying position, we have to subtract the outcome from 1 (i.e., use 1 − *P*) to get the probability that the hoverfly was “flying”.

### Statistics

All statistical analyses were done with Prism version 7.0d (GraphPad Software, USA). Before rejecting the null hypothesis (*p* < 0.05) the data were checked for normality using D’Agostino and Pearson normality test. Where the data were normally distributed we did unpaired *t* tests when the standard deviation was similar, or Welch’s *t* test where it was not. Where the data were not normally distributed we did a Mann–Whitney test. In all cases, this was followed with Bonferroni correction for multiple comparisons (two for the ground photos, 12 for the panoramic photos).

## Results

**Fig. 1:**
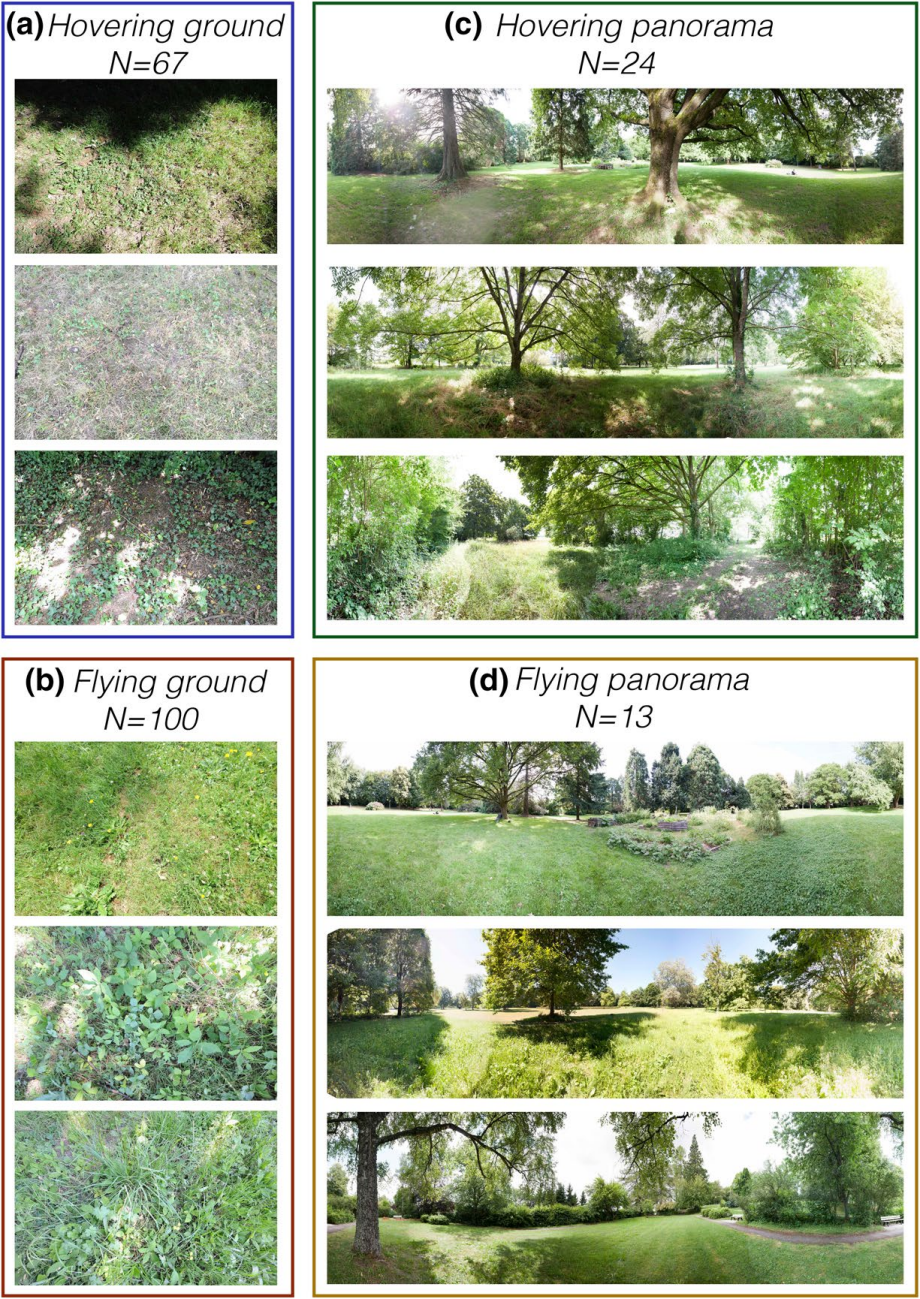
Photos taken where hoverflies were hovering or flying. **a** Example photos of the ground over which *Episyrphus* were hovering. Each photo was taken approximately 1 m above the ground. **b** Example photos of the ground over which *Episyrphus* were flying. **c** Example panoramic photos taken from the viewpoint of hovering *Episyrphus*. The panorama has been merged in Photoshop for display purposes. **d** Example panoramic photos seen from the viewpoint of a flying *Episyrphus*. The panorama has been merged in Photoshop for display purposes. All photos have been scaled for printing purposes

To quantify image statistics in locations where the marmalade hoverfly *Episyrphus balteatus* performs different behaviors, we took photos of the ground and the surrounding panorama after identifying freely behaving animals in the field. We first identified locations where male *Episyrphus* were hovering, and as comparison, took photos where *Episyrphus* were observed to be flying without apparently stopping for feeding or hovering. Since image statistics depend on the distance to the scene (Torralba and Oliva 2003) we took all photos of the ground from 1 m height to get comparable measures (hovering examples, Fig. 1a; flying examples, Fig. 1b). The panoramic surrounds were created by taking 11–13 evenly spaced photos centered on the hoverfly’s position at the time of observation (hovering examples in Fig. 1c, flying examples in Fig. 1d), also from 1 m height. Using 2-dimensional photographs, we focus our analysis on the spatial information.

### The ground under hovering hoverflies has higher slope constant and entropy

**Fig. 2:**
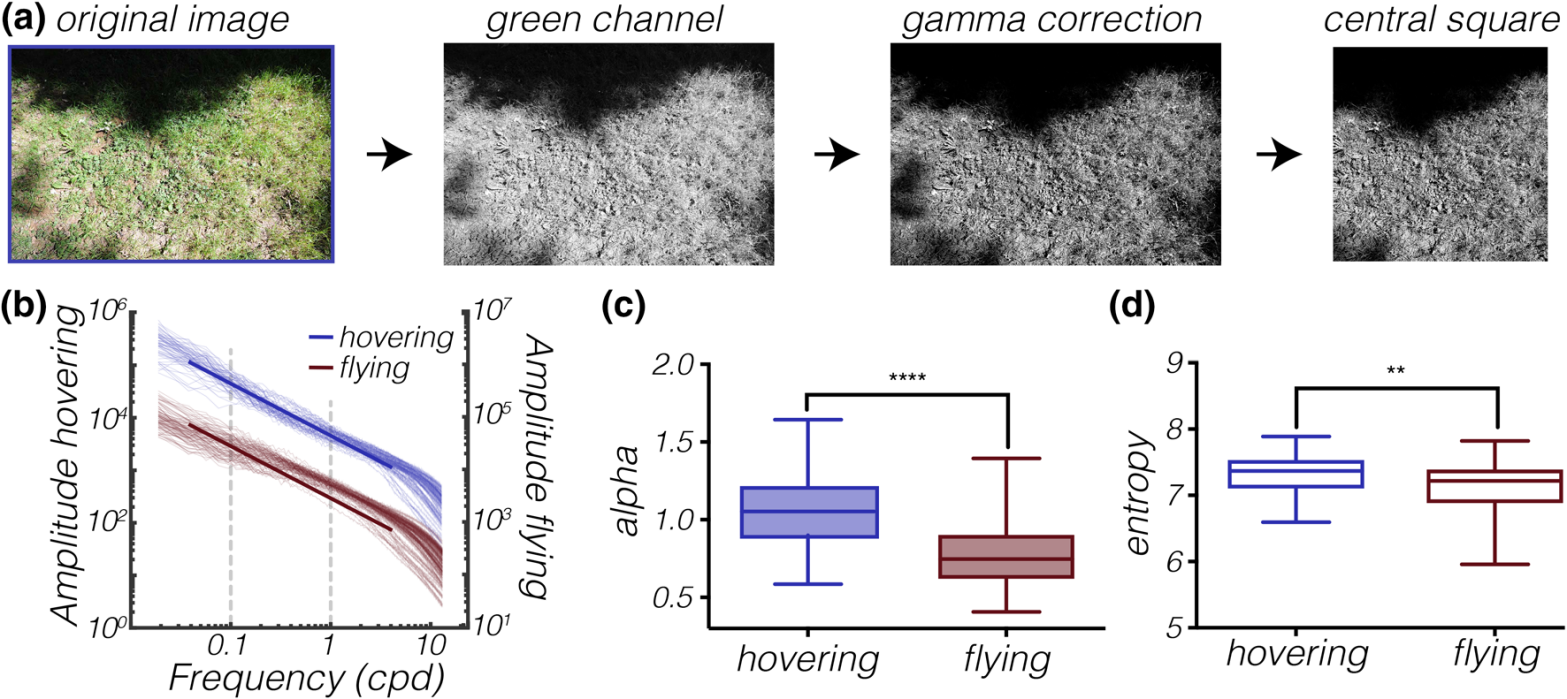
Ground photos from the viewpoint of hovering hoverflies have higher slope constant and entropy. **a** Example photo of the ground over which a male *Episyrphus* was hovering. The photo was taken approximately 1 m above the ground, using the camera’s RAW (NEF) format setting. Using Matlab, we extracted the green channel of the photo, performed an inverse gamma correction, and cropped the image to its central square before further analysis of the slope constant (alpha) and the entropy. **b** The rotationally averaged amplitude spectrum of photos of the ground over which hoverflies were hovering (blue, *N* = 67) or flying (red, *N* = 100). The fat lines indicate a slope constant of 1, i.e., a perfect power law. The dashed lines show the part of the spectrum used for calculation of the slope constant (alpha). **c** The average slope constant (alpha) of photos of the ground was significantly higher from the viewpoint of hovering hoverflies (blue, *N* = 67) than the viewpoint of flying hoverflies (red, *N* = 100; Mann–Whitney test, *p* < 0.0001). **d** The average entropy of photos of the ground was significantly higher from the viewpoint of hovering hoverflies (blue) than the viewpoint of flying (red) hoverflies (Welch’s *t* test, with Bonferroni correction, *p* = 0.0076). In panels *c* and *d* the central mark of each boxplot shows the median, the edges of the box the 25th to 75th percentiles, and the whiskers extend from the minimum to maximum of the data

A qualitative comparison between the example photos of the ground over which *Episyrphus* was hovering (Fig. 1a) or flying (Fig. 1b) suggests that there might be some interesting differences in image statistics. To investigate this, we took each original RAW (NEF) format photo and converted the RGB-image to greyscale by extracting the green channel, performed an inverse gamma correction, and cropped the rectangular photo to its central square (Fig. 2a).

We calculated the rotationally averaged amplitude spectra (Dyakova and Nordström 2017) of all the photos of the ground over which the hoverflies were hovering (*N* = 100, blue, Fig. 2b) and all the photos of the ground over which the hoverflies were flying (*N* = 67, red, Fig. 2b). If the amplitude spectrum of an individual image follows a perfect power law, with a slope constant of 1, it will follow the fat lines (Fig. 2b). We calculated the slope constant (alpha) for each image, and found that this was significantly higher in photos of the ground over which hoverflies were hovering (blue data, *p* < 0.0001, Mann–Whitney test, with Bonferroni correction for multiple comparisons, Fig. 2c) compared with where they were flying (red data, Fig. 2b). In addition, we found that whereas the alpha values of the ground over which hoverflies were hovering (blue data, Fig. 2c) were similar to reported values for natural scenes (Tolhurst et al. 1992; Dyakova and Nordström 2017), the alpha values of the ground over which hoverflies were flying (red data, Fig. 2c) were considerably lower.

We next compared the entropy in the same photos and found that the photos of the ground over which the hoverflies were hovering (blue data, Fig. 2d) had significantly higher entropy (*p* = 0.0076, Welch’s *t* test, with Bonferroni correction for multiple comparisons) than the photos of the ground over which the hoverflies were flying (red data, Fig. 2d). Lower entropy means that a photo contains more uniform textures (Ruderman and Bialek 1994; Mirzaei et al. 2013).

### The panoramas surrounding hovering hoverflies have lower slope constant

**Fig. 3:**
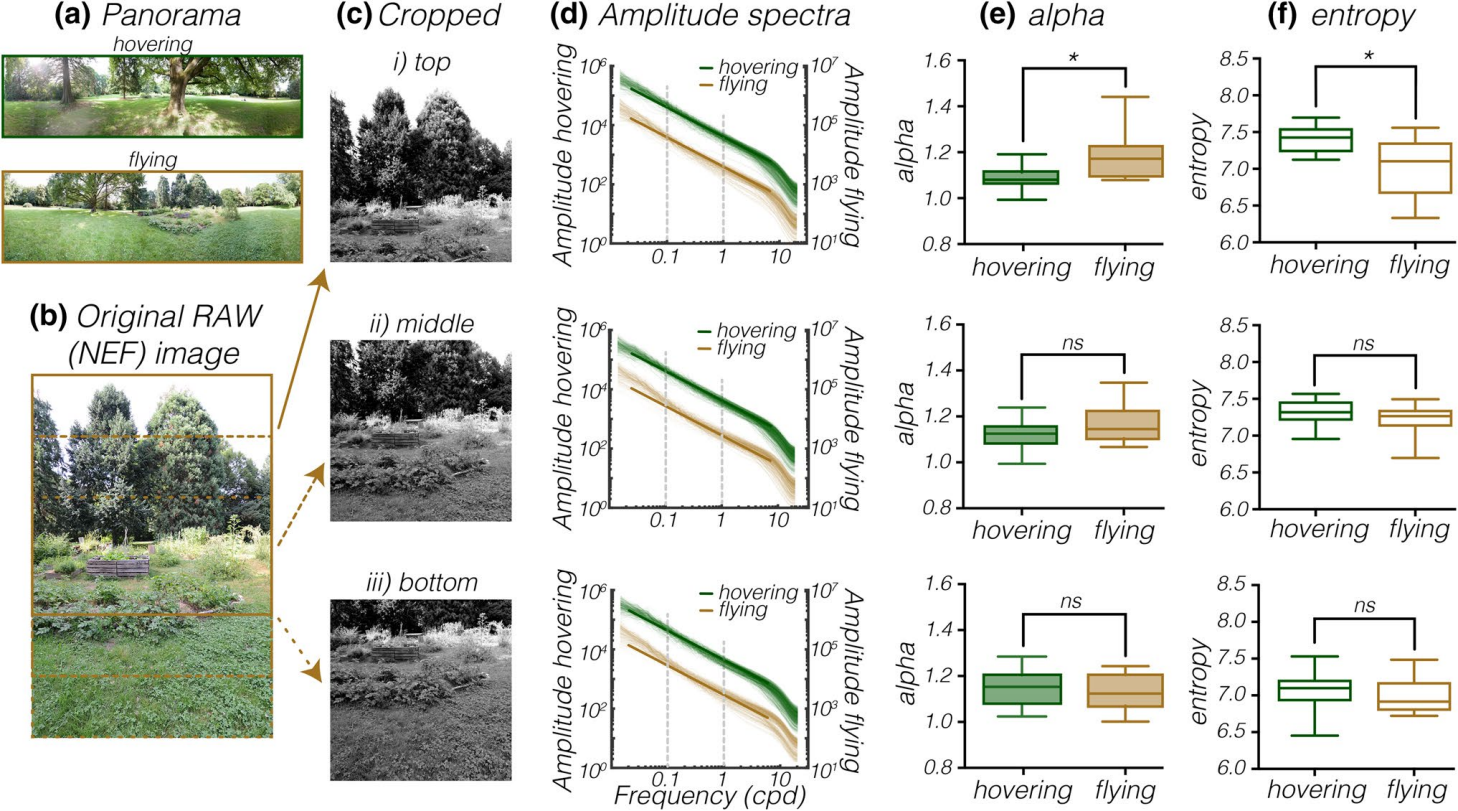
Panoramas surrounding hovering hoverflies have lower slope constant and higher entropy. **a** Example: merged panoramas surrounding a hovering (green frame) or flying (gold) male *Episyrphus*. The panoramas were merged in Photoshop for display purposes only. **b** An example of the photos taken surrounding freely behaving hoverflies. The outlines indicate the top, middle, and bottom segments. **c** The (i) top, (ii) middle and (iii) bottom segments after the green channel were extracted and the picture gamma corrected. **d** The rotationally averaged amplitude spectrum of photos of the ground over which hoverflies were hovering (green, *N* = 24) or flying (gold, *N* = 13). The fat lines indicate a slope constant of 1, and the dashed lines the part of the spectrum used for calculation of the slope constant (alpha). **e** The average slope constant (alpha) of the panoramas surrounding hovering hoverflies (green) compared with the slope constant surrounding flying hoverflies (gold). The top part of the photos showed a significant difference (Mann–Whitney test, with Bonferroni correction, *p* = 0.0102), but the middle and the bottom segments showed no significant differences (both unpaired *t* test, with Bonferroni correction). **f** The average entropy of the panoramas surrounding hovering hoverflies (green,) compared with the entropy surrounding flying hoverflies (gold). The top part of the photos showed a significant difference (Welch’s *t* test, with Bonferroni correction, *p* = 0.0302), but the middle (Welch’s *t* test) and the bottom segments showed no significant differences (unpaired *t* test). In **d** and **e** the central mark of each boxplot shows the median, the edges of the box the 25th–75th percentiles, and the whiskers extend from the minimum to maximum of the data

The image statistics of the ground (Fig. 2) can be highly affected by the surrounding area, where trees and foliage may cast shadows on the ground, or create sun shafts, in which *Episyrphus* hoverflies are known to hover (Alderman 2008). We therefore next quantified the same two image statistics of the surrounding panoramas (hovering and flying examples, Fig. 3a). For this purpose, we extracted three overlapping segments from each of the individual photos (Fig. 3b) that were taken to create a 360° view around each freely behaving hoverfly. We extracted the green channel and did a gamma correction of the top, middle and bottom elevation segments (Fig. 3c). We calculated the rotationally averaged amplitude spectrum for the hovering (green data, Fig. 3d) and flying (gold data, Fig. 3c) surrounds. We found that the alpha was significantly lower (*p* = 0.0102, Mann–Whitney test, followed by Bonferroni correction for multiple comparisons) in the top segments of the panoramas surrounding hovering hoverflies (green data, Fig. 3e*i*) compared with flying hoverflies, but there were no significant differences in the middle and lower parts of the panoramas (Fig. 3e*ii, iii*). Note that for the ground photos we saw the opposite effect, where flying was associated with a lower alpha (red data, Fig. 2c). The alpha values of the panoramas surrounding both hovering and flying hoverflies were within the range typically described for natural scenes (e.g., Tolhurst et al. 1992; Dyakova and Nordström 2017).

We next quantified the entropy for the top, middle and bottom elevations of the panoramas, and found that the top part of the hovering panoramas had higher entropy (green data, Fig. 3f*i*) than panoramas surrounding flying hoverflies (gold data, Fig. 3f*i*, Welch’s *t* test, with Bonferroni correction for multiple comparisons). However, there were no significant differences when comparing the middle and bottom elevations of the panoramas (Fig. 3f*ii, iii*). As entropy is a measure of variability (Ruderman and Bialek 1994; Mirzaei et al. 2013), this suggests that panoramas surrounding flying hoverflies contain areas with more uniform texture.

### A hovering hoverfly is surrounded by a more symmetrical scenery

**Fig. 4:**
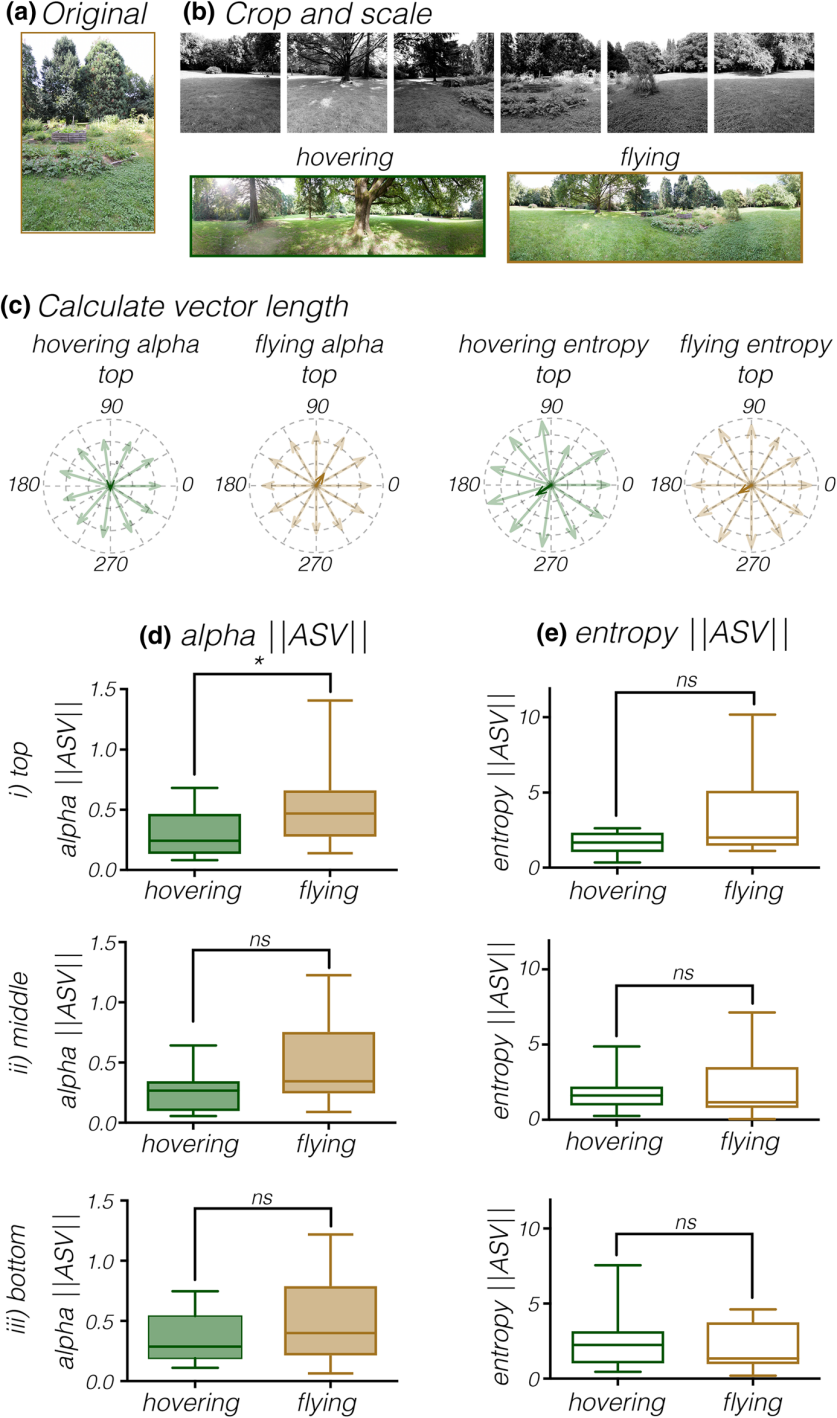
The surroundings around a hovering hoverfly are more symmetrical. **a** To calculate the symmetry of the alpha values surrounding hovering and flying hoverflies we started with the original RAW (NEF) photos. **b** We extracted the greyscale image, performed a gamma correction and cropped the top, middle, and bottom segment of each photo. **c** The alpha or entropy of these images was then placed in a virtual cylinder surrounding the hoverfly. We next calculated the symmetry vector (||ASV||) across the panorama, where a value of 0 indicates that all values across the panorama are identical. The examples show the individual alpha and entropy values, and the resulting symmetry vectors, for the top segments of the panoramas in **b**, hovering in green and flying in gold. **d** The length of the alpha ASV (||ASV||) of the top part of the panoramas surrounding hovering hoverflies is lower than the length of the ASV of panoramas surrounding flying hoverflies (unpaired nonparametric Mann–Whitney test, with Bonferroni correction for multiple comparisons, *p* = 0.0444), but there was no significant difference for the middle or lower parts (both Welch’s *t* test, with Bonferroni correction for multiple comparisons). **e** The length of the entropy ASV (||ASV||) of the panoramas surrounding hovering hoverflies was not significantly different from the panoramas surrounding flying hoverflies (unpaired nonparametric Mann–Whitney test, with Bonferroni correction for multiple comparisons). In **d** and **e** the central mark of each boxplot shows the median, the edges of the box the 25th–75th percentiles, and the whiskers extend from the minimum to maximum of the data

To investigate whether the apparently more uniform texture associated with flying hoverflies (Fig. 3f*i*), means that the surround is also more symmetrical, we used a variant of the average landmark vector (ALV) model (Lambrinos et al. 2000), called the Average Skyline Vector model (Hafner 2001; Müller et al. 2018). For this, we used the same image preparation (Fig. 4a, b), but we now placed the extracted values (alpha or entropy) from each segment at each elevation (top, middle or bottom) evenly distributed within a virtual cylinder (Fig. 4c). This gave us vectors with directions evenly distributed across the 360° field of view, and magnitudes given by the corresponding alpha or entropy value (Fig. 4c). We next summed all of the vectors to form the Average Skyline Vector (ASV). Doing this means that if all the individual vectors (faded colors, Fig. 4c) are identical, the corresponding Average Skyline Vector (solid colors, Fig. 4c) will be zero. This therefore allows us to use the length of the ASV as a measure of how symmetrical a panorama is.

In our hovering and flying examples we found that the length of the alpha ASV was lower for the top panoramas surrounding a hovering hoverfly than those surrounding a flying hoverfly (Fig. 4d*i*, Mann–Whitney test, with Bonferroni correction for multiple comparisons, *p* = 0.0444), suggesting that the alpha values are more symmetrically distributed. However, there was no significant difference for the middle and lower parts of the panoramas (Fig. 4d*ii, iii*), nor for the entropy values (Fig. 4e).

### Hoverfly behavior can be predicted from ground photos

**Fig. 5:**
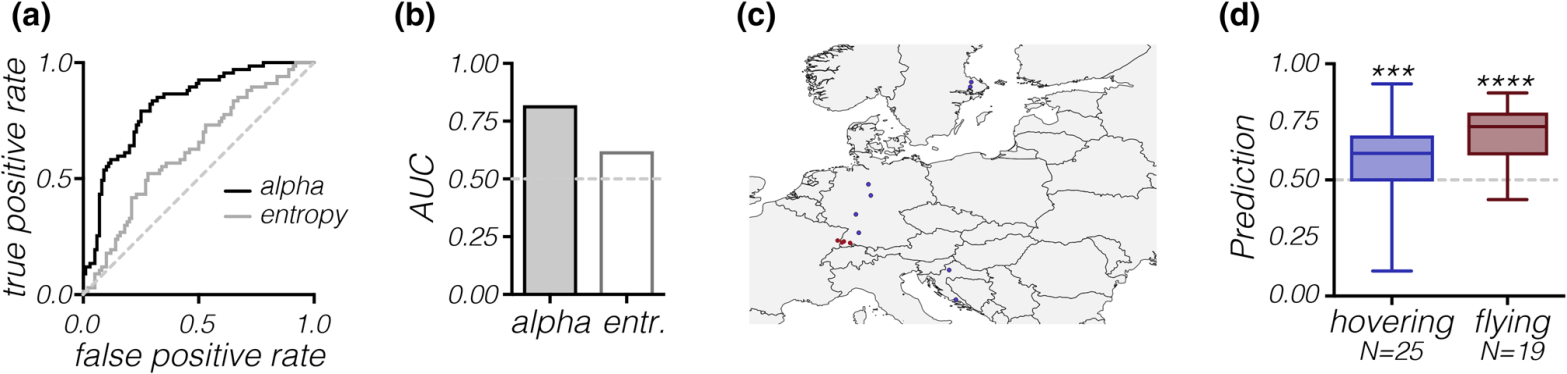
A logistic-regression model based on the ground photos can be used to predict behavior. **a** The ROC curve shows the true positive rate as a function of the false positive rate for a logistic-regression model based on the alpha (black) or the entropy from the photos of the ground over which hoverflies were either flying or hovering (data from Fig. 2c, d). The dashed line indicates a model that performs no better than chance. **b** The area under the ROC curve (data in panel a). The dashed line indicates a model that performs no better than chance. **c** The red symbols denote where the photos were taken that were used to create the logistic-regression model. The blue symbols mark where we obtained the independent photos that were used to test the model. **d** The probability of predicting the correct behavior based on image alpha of independent photos of the ground. The dashed line indicates a prediction that is no better than chance level (0.5, Wilcoxon signed-rank test)

We found the biggest difference between photos taken where hoverflies were hovering and flying in the photos of the ground (Fig. 2). Can these differences be used to predict *Episyrphus* behavior? To investigate this, we created two logistic-regression models based on image alpha and entropy (i.e., using the data in Fig. 2c, d). Logistic-regression models are used when the dependent parameters are categorical (in this case hovering vs flying) rather than continuous (Tolles and Meurer 2016). We created one model based on the alpha (black, Fig. 5a), and one based on the entropy (grey, Fig. 5a), where the resulting receiver operating characteristic (ROC) curves show the true positive rate (also called sensitivity) as a function of the false positive rate (also called 1-specificity) and illustrate how well each model discriminates between the two outcomes. If a given model is no better than chance, its resulting ROC curve will follow the diagonal (dashed line, Fig. 5a), but the closer the ROC curve is to the upper, left-hand corner of the graph, the closer the logistic-regression model is to having perfect discrimination power (Hajian-Tilaki 2013; Sainani 2014). The area under the curve (AUC) can be used to summarize the ROC curves, suggesting that image alpha is a better predictor (Fig. 5b).

As the logistic-regression model based on alpha provided the better model (Fig. 5a, b), we used its output to predict the expected behavior in an independent set of photos. The photos that were used to create the model were taken in Germany (red, Fig. 5c), whereas the photos used for validating the model were taken in different locations, in a different year (Germany, Croatia and Sweden, blue, Fig. 5c). There is thus no chance that there could be any overlap in the two data sets that could bias the prediction. If the prediction would be no better than chance, we would expect it to be 0.5 (dashed line, Fig. 5d). However, we found that the model was able to predict the correct behavior significantly better than chance (Wilcoxon signed-rank test, *p* = 0.0088 and *p* < 0.0001 for hovering and flying, respectively, Fig. 5d).

## Discussion

The use of image statistics allows us to perform quantitative comparisons of photos of different scenes. Here, we show that photographs of the ground over which hoverflies were hovering have higher slope constant (alpha) of the amplitude spectrum and entropy than those taken where hoverflies were flying (Fig. 2). We also show that the surrounding panoramas have lower alpha (Fig. 3e*i*) and asymmetry (Fig. 4d*i*), but higher entropy (Fig. 3f*i*), where hoverflies were hovering compared to where they were flying. Finally, we show that the alpha of the ground photos could be used to predict the behavior in an independent data set (Fig. 5d).

### Image statistics

Image statistics are used to quantify images, and provide measures that allow comparisons (Pouli et al. 2011). There are many different image statistics available, filling entire textbooks, from the color and contrast of an image, to second- and higher order correlations. To provide an initial understanding of the image statistics that may be important for hoverflies, we chose to focus on two measures. The first one is the slope constant of the amplitude spectrum, as neurons in the hoverfly brain and descending nerve cord have been shown to be tuned to naturalistic spectra (Dyakova et al. 2015; Nicholas et al. 2018). In addition, we quantified entropy, as this provides a measure of variation (Daugman 1989) and our initial impression was that this differed substantially between habitats. This is not to suggest that these are the only image parameters that may matter to a hoverfly, but they provide a starting point for understanding habitat differences.

The slope constant (alpha) of the rotationally averaged amplitude spectrum across a wide range of naturalistic scenes show a Gaussian distribution with a peak around 1–1.2 (e.g., Tolhurst et al. 1992; Field 1993). In line with this, we found that the slope constant (alpha) of the surrounding panoramas had a mean around 1.12 when the hoverflies were hovering and 1.16 when they were flying (Fig. 3e). Previous work found that the slope constant in panoramic surrounds depends on the elevation (Schwegmann et al. 2014a), which differs from our findings (Fig. 3e). However, our separation into different elevations (Fig. 3c) provided much larger areas of overlap than Schwegmann et al. (2014a), which may explain the disparity.

Surprisingly, we found that the slope constant of the photos of the ground over which the hoverflies were flying was strikingly low, with a mean of 0.78 (Fig. 2c), much lower than what is normally reported for natural scenes (e.g., Tolhurst et al. 1992; Dyakova and Nordström 2017). Close-up photos often have higher slope constants (Graham and Redies 2010), but this does not explain our finding as the photos of the ground over which hoverflies were hovering, taken from the same height, had more typical slope constants (mean of 1.06, blue data, Fig. 2c). Indeed, all ground photos (Fig. 1a, b) were taken from the same height and viewing angle. One notable difference is that the photos of the ground over which the hoverflies were flying contained less shadows and low-frequency luminance changes (Fig. 1b) compared with where they were hovering (Fig. 1a). In contrast, the ground over which the hoverflies were flying appears to contain more high spatial-frequency texture (Figs. 1b, 2b), which could decrease the slope constant.

Whereas the power law is a good collective description, not all individual images follow it, and extracting the slope constant may fail for individual images (Pouli et al. 2010). Extracting alpha may be particularly difficult at low frequencies. We extracted alpha between 0.1 and 1 cpd, as this is most consistent with the literature and with the hoverfly optics (for justification, see Dyakova and Nordström 2017), thus avoiding the low-frequency part of the spectrum. However, a closer inspection of the amplitude spectra suggests that whereas the slope for the hovering photos remains similar up to about 6 cpd, the slopes for the flying images depend on the part of the spectrum examined (Figs. 2b, 3c). For example, the flying ground photos appear to have increased amplitude around 1 cpd compared with the hovering ground photos (Fig. 2b). Determining what part of the spectrum is most important to hoverfly vision thus needs to be elucidated in future electrophysiological and behavioral work.

In general, we found that the entropy was lower where the hoverflies were flying (Figs. 2d, 3e), indicating that the flying surrounds contained more areas of uniform texture. However, this does not imply that the flying surrounds were more symmetrical, as we found no significant differences in the ASV (Fig. 4e). To calculate entropy here we used the relative ratio of each greyscale value (Daugman 1989), however, there are other measures in the literature that take the relative orientation into account (Redies et al. 2017). While entropy has been used to understand human eye movements when viewing scenes (Hansen and Essock 2005; Renninger et al. 2007), it is largely understudied when it comes to fly vision.

### Neural coding

Our findings are important as they describe some relevant image statistics of the natural environment for different behaviors in freely behaving animals. This has previously been done for human observers (Parraga et al. 2000; Frazor and Geisler 2006), and for flying blowflies (Schwegmann et al. 2014b), but not for hoverflies. These results can in the future be used when designing naturalistic stimuli to understand the neural coding of visual neurons (Dyakova et al. 2015; Nicholas et al. 2018) or when quantifying visual behaviors (Goulard et al. 2015; 2016). Indeed, both vertebrate and fly peripheral visual systems seem to be tuned to naturalistic contrast distributions and to slope constants around 1 (van der Schaaf and van Hateren 1996; van Hateren 1993; Barlow 1961). More centrally, vertebrate cortical neurons appear to be optimal for coding naturalistic stimuli (Simoncelli and Olshausen 2001; Field 1987; Parraga et al. 2000). Furthermore, human subjects are good at guessing the original slope constant of manipulated images (Field and Brady 1997; Dyakova et al. 2019) and artists appear to have implicit knowledge of the amplitude spectrum, as the slope constant of most artwork is close to 1 (Graham and Redies 2010). In addition, human observers find images with naturalistic alphas as more pleasant to view (O’Hare and Hibbard 2013).

In the hoverfly visual system, a higher order visual neuron in the optic ganglion, the lobula plate, is inhibited by stationary images, which could serve a role in indicating perfect hovering (De Haan et al. 2013). Later work showed that the inhibition is strongest when the slope constant is close to 1 (Dyakova et al. 2015), which is also the alpha value that we found for scenery experienced during hovering (Figs. 2c, 3d). Closer to the behavioral output, in the hoverfly descending nerve cord, neurons that are tuned to self-generated optic flow respond strongest to images with slope constants close to 1 (Nicholas et al. 2018). However, we found that the ground photos over which the hoverflies were flying had a much lower alpha value (Fig. 2c), something that will be of importance when designing stimuli for future work. Nevertheless, the surrounding panorama had a more naturalistic slope constant (Fig. 3e).

Recent work on the neural computations involved in the generation of sensitivity to visual motion has suggested the ON–OFF asymmetries in the peripheral pathways are optimized for naturalistic input (e.g., Leonhardt et al. 2016; Fitzgerald and Clark 2015). Understanding and quantifying the relevant natural environment, and how this may differ between behaviors, is thus fundamental for interpreting the response tuning of visual neurons. Our results presented here provide such data, at least for hoverflies in the spatial domain.

### Behavioral relevance

Importantly, our work only took spatial statistics into account, which is obviously a simplification, especially when analyzing the statistics surrounding flying hoverflies. Previous work investigating the temporal luminance changes a flying insect experiences, suggests that these follow a similar power law as the spatial luminance spectrum (van Hateren 1997). Furthermore, early vision appears to whiten the spectrum in time, just like it does in space (van Hateren 1992). More recent work has shown that fly photoreceptors can extract more information from natural time series compared with artificial, white noise stimuli (Song and Juusola 2014; Juusola and Song 2017). In the future it will be interesting to do a similar analysis of the time series experienced by hoverflies when they are hovering, compared with where they are flying, or performing other behaviors, and to record neural responses to these. As more hoverfly behavioral data becomes available, this aim will become more achievable (Thyselius et al. 2018; Goulard et al. 2015; Geurten et al. 2010). Indeed, there is a strong correlation between the temporal and spatial-frequency spectra (Dong and Atick 1995), and as a flying insect will be experiencing higher temporal frequencies than a hovering fly, this could affect the influence of the low alpha in the spatial-frequency spectrum.

Based on the photographs taken in different habitats, we discovered profound differences in the 2D image statistics at places where hoverflies were found to hover compared with those encountered during cruising flight. While this finding is of great functional relevance with respect to habitat choice, the data set on which it is based does not allow us to draw conclusions with regard to the environmental information and, in particular, the temporal cues, nor the 3 dimensional spatial cues that are computed by hoverflies from the retinal image flow during hovering and cruising flight, respectively. To address this complex and interesting issue, movie sequences (Schwegmann et al. 2014a, b), rather than photographs, would be required as these could reflect the peculiar flight dynamics of hoverflies in the different behavioral situations and habitats.

Previous work has shown that humans fixate at a given location within a natural scene for only 200–300 ms before shifting the gaze (Frazor and Geisler 2006), and that the local luminance and contrast of the part of the image projecting onto the fovea may change rapidly between these saccades. In blowflies it has been shown that the slope constant does not change much during forward translation through open scenes, but may change more dramatically through forward translation through a forest scene, and much more during yaw rotations (Schwegmann et al. 2014b). As our definition of flying was a forward translating hoverfly, and as these were mostly observed in more open scenes (Fig. 1b, d), we find it likely that the alpha did not change much during this behavior (Schwegmann et al. 2014b).

Hoverflies tethered on a trackball set-up appear to perform the strongest optomotor response when stimulated with images with slope constants close to 1.2 (Dyakova et al. 2015). Hoverflies have a wide field of view, and relatively good spatial resolution for insects (Collett and Land 1975; Straw et al. 2006). We thus find it likely that they would have a clear view of both the ground over which they were behaving (Fig. 1a, b), and of the surrounding scenery (Fig. 1c, d). Indeed, the only part of the surround where their visual field of view is restricted is the part obscured by the body. Hovering behavior (Alderman 2008) is usually observed where the sunshine passes through the foliage of the surrounding trees (Fig. 1c), which creates light shafts in the air and high-contrast shadow patterns on the ground (Fig. 1a). In contrast, the panoramas taken from the flying viewpoint display more open space (Fig. 1b, d). Such open scenes have previously been described to have higher alpha than forest scenes (Schwegmann et al. 2014b), which we also saw, at least at higher elevations (Fig. 3e*i*).

All our photos were taken at a similar time of the day, and tended to be taken in similar weather, as hoverflies are preferential to nice sunny days (Alderman 2008). Previous work suggests that at least the amplitude spectrum is relatively robust against different weather conditions and time of day (van der Schaaf and van Hateren 1996). In future work it will be interesting to investigate the influence weather and time of day have on different image parameters. However, non-visual parameters, such as temperature and wind (Gilbert 1985; Ottenheim 2000), also play a strong role in habitat selection. It will additionally be interesting to extract image parameters from a much wider range of behaviors.

### Concluding remarks

Previous work in different insects has shown that the texture presented in the ventral and lateral field of view is important for flight control (Linander et al. 2017, 2018; Portelli et al. 2011; Straw et al. 2010). Our results suggest that it would be interesting to do similar experiments in *Episyrphys* hoverflies, by e.g. placing them in a flight tunnel and manipulating the slope constant of the texture presented on the walls and the ground. Will a low slope constant on the ground (Fig. 2c), together with a slightly increased (Fig. 3e), asymmetrical slope constant (Fig. 4d) and reduced entropy (Fig. 3f) on the walls induce the hoverflies to favor flying over hovering?
